# Severe hypoxia selects hematopoietic progenitors with stem cell potential from primary Myelodysplastic syndrome bone marrow cell cultures

**DOI:** 10.18632/oncotarget.24302

**Published:** 2018-01-24

**Authors:** Erico Masala, Ana Valencia-Martinez, Serena Pillozzi, Tommaso Rondelli, Alice Brogi, Alessandro Sanna, Antonella Gozzini, Annarosa Arcangeli, Persio Dello Sbarba, Valeria Santini

**Affiliations:** ^1^ MDS UNIT, Hematology, AOU-Careggi University Hospital, Department of Experimental and Clinical Medicine, Università degli Studi di Firenze, Florence, Italy; ^2^ Department of Experimental and Clinical Medicine, Università degli Studi di Firenze, Florence, Italy; ^3^ General Laboratory, AOU-Careggi, Florence, Italy; ^4^ Cellular Therapy and Transfusional Medicine Unit, Hematology, AOU-Careggi University Hospital, Florence, Italy; ^5^ Department of Medical Biotechnologies, Università degli Studi di Siena, Siena, Italy; ^6^ Department of Experimental and Clinical Biomedical Sciences “Mario Serio”, Università degli Studi di Firenze, Florence, Italy

**Keywords:** MDS, stem cells, hypoxia, high risk MDS, mice transplantation

## Abstract

Myelodysplastic Syndromes (MDS) are clonal neoplasms where stem/progenitor cells endowed with self-renewal and capable of perpetuating the disease have been demonstrated. It is known that oxygen tension plays a key role in driving normal hematopoiesis and that hematopoietic stem cells are maintained in hypoxic areas of the bone marrow (BM). Hypoxia could also regulate leukemic/dysplastic hematopoiesis. We evaluated the stem cell potential of MDS cells derived from the BM of 39 MDS patients and selected under severe hypoxia. MDS cells rescued from hypoxia-incubated cultures were subjected to stem and progenitor cell assays *in vitro,* as well as to hematopoietic reconstitution assay in NOD-SCID mice. Incubation in severe hypoxia of cells explanted from MDS patients selected a cell subset endowed with stem cell potential, as determined *in vitro*. This occurred only from the BM of patients classified as IPSS low/INT-1 risk. Transplantation into NOD-SCID mice confirmed using an *in vivo* model that severe hypoxia selects a cell subset endowed with stem cell potential from bone marrow mononuclear cells (BMMC). derived from patients belonging to the IPSS low/int-1 risk group. Data here reported show that cells endowed with stem cell potential and capable of adapting to hypoxia and escaping hypoxia-induced apoptosis exist within MDS cell populations.

## INTRODUCTION

Myelodysplastic syndromes (MDS) have been shown to comprise stem/progenitor cells endowed with self-renewal and capable of perpetuating the disease [1]. The cytogenetic and genetic abnormalities found in MDS are accompanied by a number of changes of immune system and bone marrow (BM) microenvironment [2, 3], all defects which could affect the biology of MDS stem/progenitor cells and contribute to the heterogeneity of this group of diseases [4]. How stem cell regulation is altered in MDS and how many residual normal hematopoietic stem cells (HSC) are present in the various stages of these diseases is still not clear [5].

Oxygen tension plays a key role in normal hematopoietic development and stem cells niches, where HSC are hosted, are placed in the most hypoxic areas of BM [6]. In particular, severe hypoxia modulates the balance between generation of progenitors and HSC maintenance in favour of the latter, and resistance or sensitivity to severe hypoxia defines hierarchical levels within normal hematopoietic populations [7–9]. Oxygen tension is likely to control also MDS hematopoiesis. For instance, MDS cells cultured in 1–3% O_2_ were shown to exhibit an increased colony formation efficiency [10]. The capacity of adaptation of MDS stem/progenitor cells to hypoxia, as well as the majority of human tumor stem cells [11], can be crucial not only for the maintenance of disease, but also its progression, as hypoxia induces genomic instability [12].

In the study reported here, primary human BM mononuclear cells (BMMC) from MDS cases were cultured in hypoxia to test whether it were possible to select neoplastic stem/progenitor cells under conditions favouring HSC maintenance. Cells were obtained from different MDS subtypes, heterogeneous for their cytogenetic and overall clinical IPSS risk score, in order to verify the occurrence of phenotype-specific differences of stem cell potential. MDS cells rescued from hypoxia-incubated cultures were subjected to colony formation ability (CFA) and culture repopulation ability (CRA) assay, the latter being a stem cell assay *in vitro*, as well as to repopulation assay in NOD-SCID mice [13, 14]. The results obtained suggest that, via incubation in hypoxia, it is possible to select relatively primitive HSC from IPSS lower-risk MDS patients.

## RESULTS

### Effects of hypoxia on the maintenance of the stem cell potential of MDS cells

**Figure 1:**
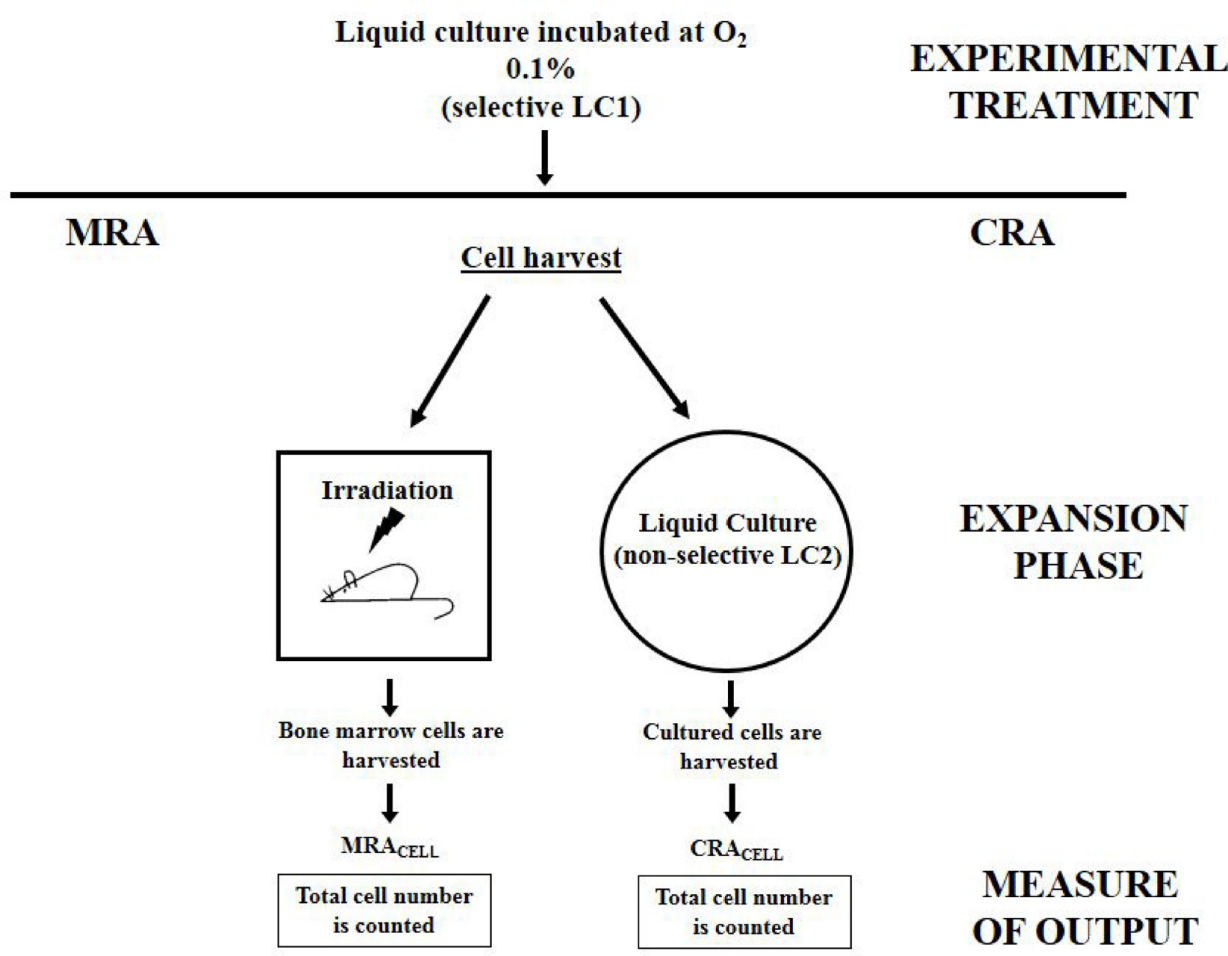
Schematic representation of CRA assay and its cognate MRA assay The CRA assay is a short-term HSC assay *in vitro* cognate to the MRA assay *in vivo*. At the end of their experimental manipulation (such as a drug treatment or, as in the experiments reported here, an incubation under selective conditions), cells are subjected to an “expansion phase” where the stem cell potential of HSC is exploited, carried out by either transplantation into syngeneic mice (Marrow Repopulation Ability) or transfer into a growth-permissive, non-selective (secondary–LC2) culture (Culture Repopulation Ability). Repopulation ability is quantified by counting the total number of cells in the bone marrow of transplantation-recipient mice or LC2, respectively (see Materials & Methods for details).

**Figure 2:**
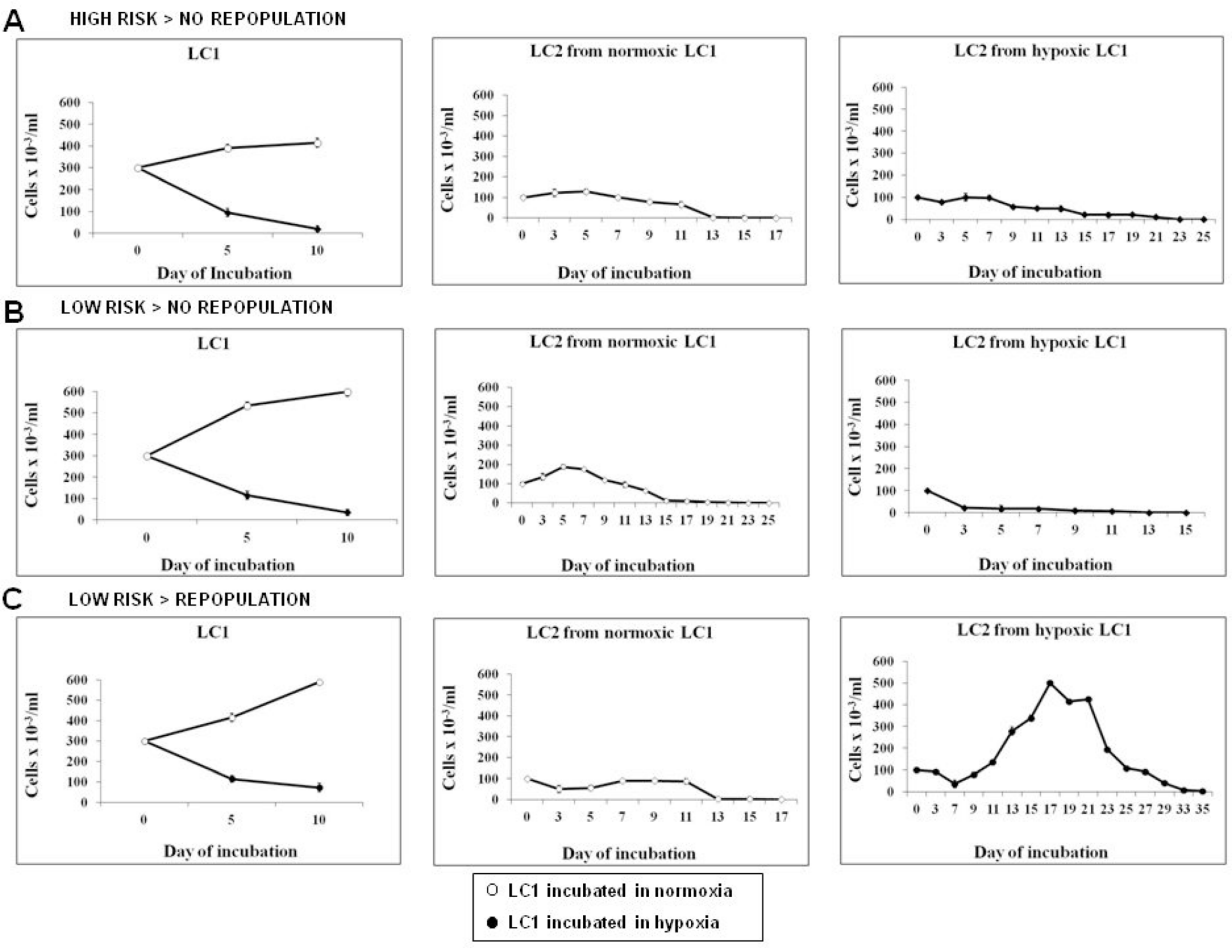
Effects of incubation in hypoxia or normoxia on total cell number or stem cell potential of MDS cells BMMC were explanted from three patients taken as examples of the three different case subsets considered (see Tables 1 and 2). The three patients were classified as: (**A**) high risk IPSS RAEB-2, (**B**) low/inte1 IPSS risk RCMD, (**C**) low/int-1 IPSS risk RA. Cells were incubated in low oxygen or in air (LC1) for 10 days (left panels) and then transferred to secondary cultures incubated in air (LC2) for the measure of stem cell potential of LC1 cells by CRA assay (cells from normoxic LC1, middle panels; cells from hypoxic LC1, right panels).

The stem cell potential of BMMC obtained from 39 MDS cases and incubated at 0.1% O_2_ or under standard conditions (“normoxia”) was estimated by the CRA assay (Figure 1). Cells were incubated in primary liquid cultures (LC1) at 0.1% O_2_ or in normoxia and then transferred to secondary cultures (LC2) always incubated in normoxia. The stem cell potential at the end of “selective” LC1 was estimated by measuring the output of “growth-permissive” LC2. Figure 2 shows examples of the three observed different outcomes. Viable cell number in LC1 decreased of at least one log at day 10–13 of culture in 0.1% O_2_, while it increased in normoxia, as expected (Figure 2A–2C, left panels). MDS IPSS high risk (RAEB-2, case #21) and IPSS low/int-1 risk (RCMD, case #7) cases did not repopulate LC2 efficiently (middle and right panels), irrespective of whether LC1 had been incubated in 0.1% O_2_ or in air (Figure 2A, 2B). On the contrary, an IPSS low/int-1 risk case classified as RCMD (case #4) was capable of a significant LC2 repopulation, but only when cells were incubated in low oxygen (Figure 2C, middle and right panels). LC2 repopulation results relative to the whole group of patients studied, reported in Table 1, can be summarized as follows:

1. IPSS high risk - BMMC → LC1 in 0.1% O_2_ or in air → no LC2 repopulation (9/9 cases);
2. IPSS low risk - BMMC → LC1 in 0.1% O_2_ or in air → no LC2 repopulation (16/30 cases);
3. IPSS low risk - BMMC → LC1 in 0.1% O_2_ or in air → LC2 repopulation (14/30 cases) only with cells from LC1 in 0.1% O_2_ (See Supplementary Figure 1A–1E). The peak values of LC2 repopulation achieved and the day of peak are reported in Table 2. The IPSS risk categories are defined in the Materials & Methods section.

**Table 1:** Overall patient characteristics

Case #	Age	Sex	WHO	IPSS	IPSS-R	BM Blast%	Karyotype	CRA
***1***	71	M	RCMD	int-1	int	0	7q-[15/20]	no
***2***	88	M	RAEB-2	high	very high	20	11q-[18/20]	no
***3***	74	F	RCMD	low	very low	0	normal	yes
***4***	48	M	RCMD	int-1	int	0	complex	yes
***5***	65	M	RCMD	low	very low	0	normal	yes
***6***	56	F	RAEB-1	int-1	int	2	normal	no
***7***	65	F	RCMD	low	very low	0	normal	no
***8***	81	F	RCMD	int-1	int	0	+8[17/20]	no
***9***	71	F	RCMD	int-1	int	0	+8[13/20]	no
***10***	78	M	RCMD	low	int	0	inv14	no
***11***	73	F	RA	int-1	int	0	t(7;12)	yes
***12***	67	F	RAEB-2	high	very high	19	complex	no
***13***	83	F	RAEB-1	high	high	8	normal	no
***14***	78	M	RCMD	int-1	Int	4	normal	no
***15***	88	M	RA	low	low	0	20q-[17/20]	yes
***16***	70	F	RAEB-1	int-1	high	8	5q-[16/20]	no
***17***	76	M	RAEB-1	int-1	int	5	normal	no
***18***	56	F	RCMD	int-1	Int	0	7q-[18/20]	no
***19***	80	F	RCMD	low	very low	0	normal	yes
***20***	71	M	RCMD	int-1	Int	0	20q-[14/20]	yes
***21***	72	M	RAEB-2	high	high	17	normal	no
***22***	70	F	RAEB-2	high	very high	20	normal	no
***23***	83	F	RAEB-2	int-2	high	16	normal	no
***24***	89	F	RA	low	low	0	normal	yes
***25***	70	M	RAEB-1	int-1	int	8	normal	yes
***26***	76	M	RA	int-1	int	5	+8[14/20]	no
***27***	82	M	RA	low	very low	0	-Y[16/20]	yes
***28***	83	F	RAEB-2	int-2	high	18	complex	no
***29***	60	M	RCMD	low	very low	0	normal	yes
***30***	63	M	RA	int-1	low	0	+8[16/20]	yes
***31***	81	M	RAEB-2	high	high	15	+8[18/20]	no
***32***	79	M	RAEB-2	high	very high	20	complex	no
***33***	72	M	RCMD	int-1	int	0	7q-[15/20]	no
***34***	78	F	5q- Syndrome	low	very low	0	5q-[18/20]	no
***35***	77	F	5q- Syndrome	int-1	very low	0	5q-[17/20]	no
***36***	69	M	RCMD	low	very low	0	normal	no
***37***	71	M	5q- Syndrome	low	very low	0	5q-[19/20]	no
***38***	83	F	5q- Syndrome	int-1	low	0	5q-[18/20]	yes
***39***	79	M	RCMD	low	very low	0	normal	yes

Among the IPSS low/int-1 risk group, 14/39 (36%) cases (dark grey) after incubation in low oxygen exhibited stem cell potential determined by culture repopulation ability (CRA) assay, while 16/30 cases did not show any CRA (light grey). Neither the cases classified as IPSS high/int-2 risk cases exhibited CRA (white).

**Table 2:** LC2 repopulation data relative to the MDS cases endowed with CRA

Case^#^	WHO	IPSS	IPSS-R	BM Blast %	Karyotype	Day of peak of LC2 repopulation	Value of LC2 repopulation at peak (cell × 10^−3^/ml)
***3***	RCMD	low	very low	0	normal	21	306
***4***	RCMD	int-1	int	0	complex	17	550
***5***	RCMD	low	very low	0	normal	19	355
***11***	RA	int-1	int	0	t(7;12)	19	498
***15***	RA	low	low	0	20q[10/20]	17	190
***19***	RCMD	low	very low	0	normal	25	275
***20***	RCMD	int-1	int	0	20q[10/20]	15	208
***24***	RA	low	low	0	normal	17	210
***25***	RAEB-1	int-1	int	8	normal	17	250
***27***	RA	low	very low	0	–Y[17/20]	15	491
***29***	RCMD	low	very low	0	normal	15	445
***30***	RA	int-1	low	0	+8[16/20]	21	244
***38***	5q-Syndrome	Int-1	low	0	5q-[18/20]	17	276
***39***	RCMD	low	very low	0	normal	19	407

The incubation day and the value of peak of LC2 repopulation for the 14/39 CRA-positive cases are reported.

CFA assays were carried out in parallel to CRA assays. GM, E and, in one case, GEM colonies were formed. Colonies were generated from the low-risk cases endowed with CRA, but not from high-risk cases or from the low-risk cases which did not show CRA (data not shown). Taken together, the data indicated that hematopoietic potential was maintained only by MDS cells derived from patients classified as IPSS low/INT-1 risk and only following incubation in 0.1% O_2_.

### Effects of hypoxia on the expression of cell surface antigens in MDS cells

**Figure 3:**
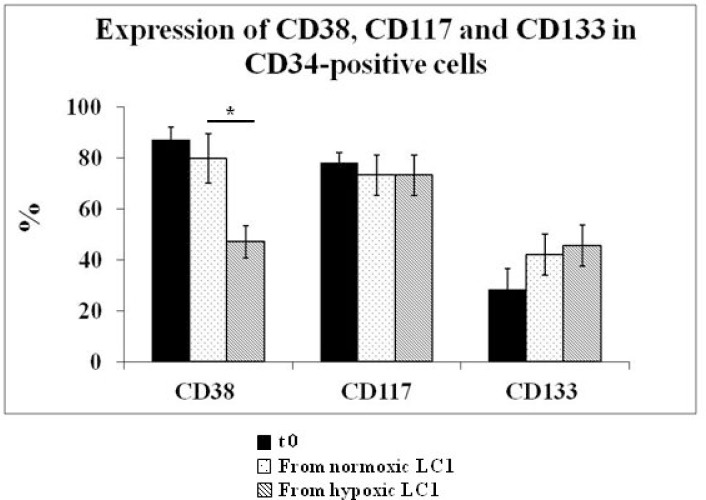
Effects of incubation in hypoxia or normoxia on the expression of CD38, CD117, CD133 in CD34-positive cells BMMC explanted from all patients listed in Table 1 were subjected to flow cytometry to measure the expression of CD38, CD117 and CD133 in CD34-positive cells at time 0 and following a 10-day incubation in hypoxia or normoxia. The significance of differences was calculated by the Student *t* tests for paired samples; ^*^ = *P* < 0.0027.

**Figure 4:**
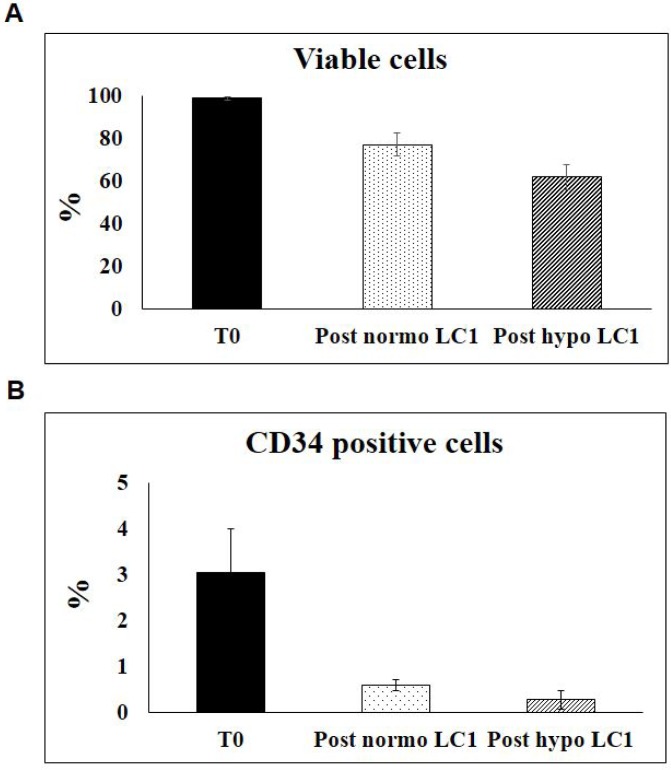
Effects of incubation in hypoxia or normoxia on the viability and the expression of CD34 in BMMC BMMC explanted from all patients listed in Table 1 were incubated with 7-Amino-Actinomycin D (7-AAD)- Viability Dye, at time 0 and following a 10–13 day incubation in hypoxia or normoxia, in order to identify the percentage o viable cells (**A**). Relative CD34 expression of viable cells was evaluated, and normalized *per* experimental point (**B**).

The expression of surface markers CD34, CD38, CD117, CD133 in BMMC from all 39 MDS cases studied was evaluated before and after incubation in 0.1% O_2_ or in normoxia (Figure 3). The percentage of viable cells as well as the relative percentage of viable CD34-positive cells decreased with respect to time 0 following either incubation condition (Figure 4). A significant difference of marker expression with respect to time 0 was observed only in cells incubated at 0.1% O_2_ and only for CD38, which was about 50% reduced. This reduction is consistent with the selection of relatively immature hematopoietic progenitor cells.

### Cytogenetic analysis

BMMC derived from 5 MDS patients (see Table 1 for complete data) with pre-identified chromosomal aberrations were subjected to FISH analysis at time 0 of culture and after incubation in hypoxia. The cases analyzed were: one RCMD with 7q- (case #18), one RCMD with 20q- (case #20), one RA with –Y (case #27), all classified as IPSS low/int-1 cases; two RAEB-2 (#28 and #32), which presented a complex karyotype, classified as IPSS high risk cases.

In all cases we observed the maintenance of equal percentages of cells with chromosomal aberrations after hypoxia incubation respect to time 0. In particular, in the RCMD case #20, characterized by 7q deletion, the percentage of cells with chromosomal aberration was 68% at time 0 and 65% following incubation in hypoxia. Identical percentages were obtained by FISH analysis of RCMD case #20. In the #27 RA case, characterized by deletion of Y, –Y cells were 65,6% at baseline and 82,4% after incubation in hypoxia; this case was one of the 14 characterized by CRA, while at the peak of LC2 repopulation (day 15 of incubation), FISH analysis showed a reduction of percentage of cells with chromosomal aberration (68,8%) respect to cell population after hypoxia incubation, as if normal cells were overgrowing. Among the RAEB-2 cases analysed, #28 and #32 showed a percentage of cells characterized by complex karyotype of respectively 66% and 60% at time 0 and 70% and 62% after incubation in hypoxia. We suppose that the cells with normal karyotype may belong to the normal residual hematopoietic progenitor cell population. Following incubation in hypoxia, cells from all cases maintained the initial chromosomal aberrations, indicating suggesting that cells resistant to/selected in hypoxia belong to the original cell clone.

### Evaluation of genes commonly mutated in MDS

We performed mutation analysis of 8 cases (seven classified as IPSS low/int-1 risk and one as IPSS high risk) by NGS at time 0, but we could not demonstrate a specific correlation between baseline number and type of mutations and CRA after incubation (data no shown).

### *In vivo* engraftment of MDS cells incubated in hypoxia

**Figure 5:**
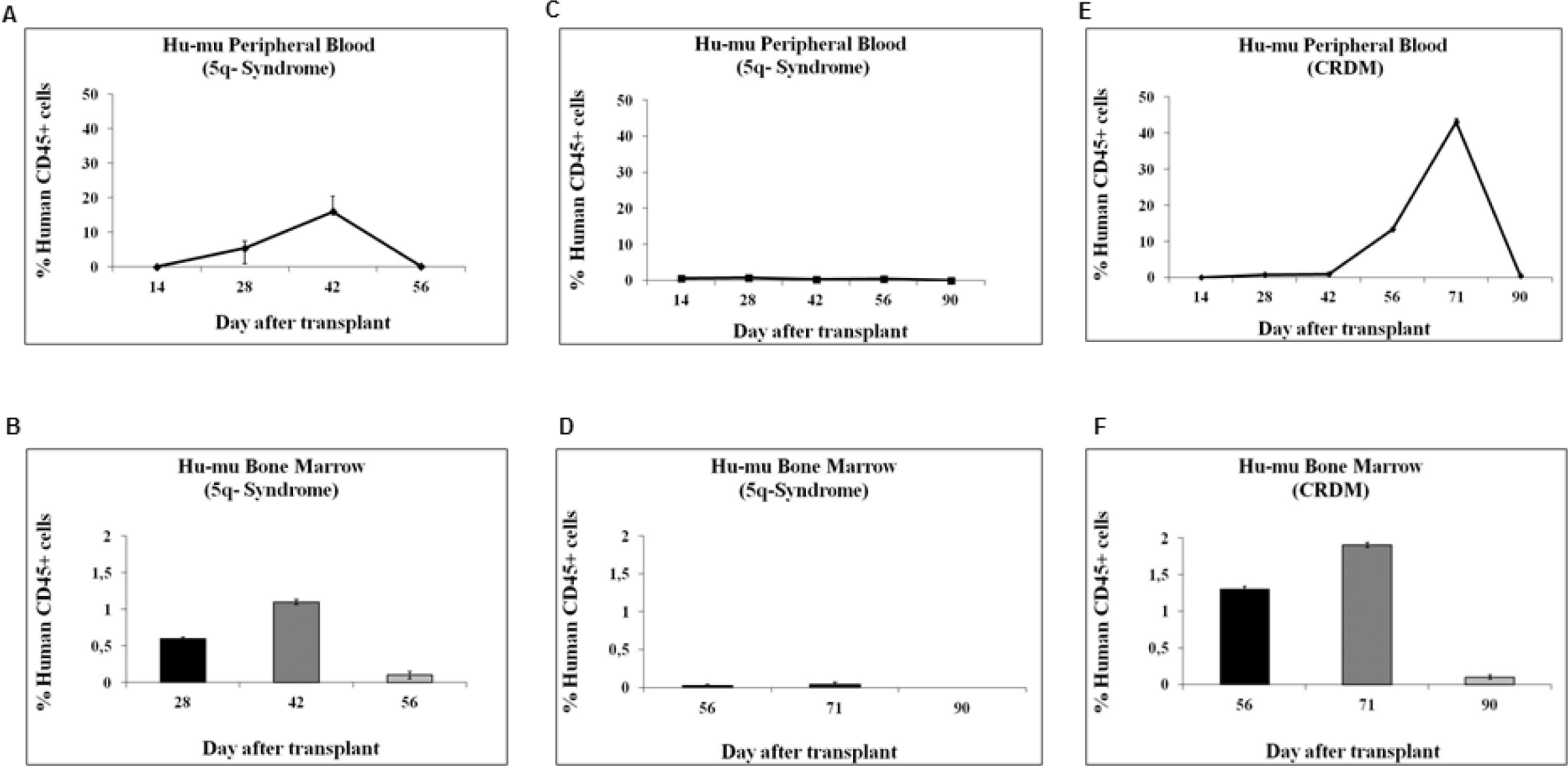
Kinetics of engraftment of BMMC derived from MDS patients in NOD/SCID mice BMMC explanted from patients classified as IPSS low/int-1 risk were incubated in low oxygen (LC1) for 10 days, and then intravenously injected into eight-week-old NOD/SCID beta 2 null mice, previously subjected to a single-dose 250cGy total body irradiation. Mice transplanted with human MDS cells were termed Hu-mu. The percentage of human CD45+ cells was determined by flow cytometry in PB or BM samples obtained from mice sequentially after transplantation. Human CD45+ cells were detected in mice transplanted with cells derived from a 5q- syndrome (**A, B**) or a CRDM (**E, F**) patient, but not in the case of a different 5q- syndrome (**C, D**).

Figure 5 shows the *in vivo* repopulation ability, measured in peripheral blood (PB) and BM of recipient mice (A, C, E and B, D, F, respectively), of cells derived from 3 different IPSS low/int-1 MDS cases. BMMC rescued from day-10 cultures incubated at 0.1% O_2_ were injected intravenously into NOD/SCID mice. A 5q- syndrome case (#38) showed a peak of human CD45-positive cells, in either PB or BM, at day 42 after transplantation (A, B); a second 5q- syndrome case (#37) failed engraftment (C, D); the CRDM case (#39) showed a peak of human CD45-positive cells, in either PB or BM, at day 71 after transplantation (E, F). We could not detect human cells in the spleens. We performed in parallel the CRA assay: case #38 (5q- syndrome) was capable of a significant LC2 repopulation too, peaking at day 17 with 2,76 × 10^5^ cells/ml; the second 5q- syndrome case (#37), that failed engraftment in mice, did not show any repopulating ability in hypoxia selected cells; CRA assay performed for CRDM case (#39) showed the repopulating capacity of hypoxia selected cells, peaking at day 19 with 4 × 10^5^ cells/ml (see Tables 1 and 2 for complete data).

## DISCUSSION

Clonal evolution of MDS increases the level of heterogeneity of these diseases and renders a homogenous approach to therapy more difficult. We learned that there are linear and branching clonal evolution paths that are patient-specific [15]. These dynamic processes most probably deeply affect the stem cell potential of individual MDS cases. The isolation of primary MDS stem cells based on biological properties, or by exploiting their capacity to propagate and proliferate in hypoxic environment, could lead to the characterization of the “stemness” level of each case and thereby to an improvement of their therapeutic targeting.

Our study showed that incubation in severe hypoxia of cells explanted from MDS patients selects a cell subset capable to escape hypoxia-induced apoptosis and endowed with stem cell potential, as determined by repopulation ability *in vitro* (CRA assay). FISH analysis showed that these cells maintain chromosomal markers typical of the original MDS clone.

Cell selection in hypoxia was possible only in a defined subgroup of MDS cases, *i.e*. derived from patients belonging to the IPSS low/int-1 risk group. Nevertheless, only 14/30 cases of this group were found endowed with repopulation ability. No chromosomal abnormality or somatic mutation detected was found to correlate with the absence or the presence of such an ability. To select under hypoxia cells exhibiting stem cell potential was impossible for IPSS high-risk cases; we reckon that hypoxia may have a non-permissive effect on dysplastic blasts. Blast morphology may indeed correspond to a hierarchical structure of cell population where stem cell potential is located within a relatively less immature cell phenotype, which is thereby hypoxia-sensitive instead of being hypoxia-resistant.

The MDS cases exhibiting measurable stem cell potential showed an apparently wide range of variations as for the time (15–25 days) and level (208–550 × 10^−3^ cells/ml of culture) of peak of LC2 repopulation. Peak time and repopulation level appeared unrelated. Wide differences among different cell populations with respect to these parameters have been observed in several studies [16–19]. A recent publication, with the exclusive analysis of a subset of IPSS low/int-1 risk MDS (5q- cases), indicated the *in vivo* existence of rare (multipotent) MDS stem cells and defined their hierarchical relationship to lineage-restricted MDS progenitor cells [20]. These results are consistent with what we observed here by CRA assay in 14 IPSS low/int-1 risk MDS cases. It is worth noting that flow cytometry analysis revealed that, when incubated in hypoxia, CD34-positive MDS cells underwent a significant reduction of CD38 co-expression with respect to normoxia-incubated controls. This reduction is well in keeping with the selection of relatively less mature hematopoietic progenitor cells, which are those able to adapt to the hypoxic environment.

Transplantation into NOD-SCID mice confirmed using an *in vivo* model that severe hypoxia selects a cell subset endowed with stem cell potential from BMMC derived from patients belonging to the IPSS low/int-1 risk group. However, these experiments could not confirm that engraftment was determined by MDS stem cells. Rather, it is possible that normal hematopoietic progenitor cell population, endowed as expected with stem cell potential and capacity of adaptation to hypoxia, did engraft, generating the CD45-positive human cells detected in PB and BM of recipient mice. It is of interest that this was possible only in IPSS low/int-1 risk cases, but not in the high risk patients. The kinetics of hematopoietic reconstitution observed in PB and BM of transplanted mice were different. This may reflect the selection of cell subsets of different hierarchical level within the stem cell compartment, due to the patient-specific clonal structure of disease. The evaluation of MDS clone burden in a higher number of cases, as well as the detection of the original mutation in the MDS cells expanded following incubation in hypoxia could shed more light onto the variables affecting the growth of hypoxia-selected primitive cells.

If *in vivo* experiments will confirm that MDS stem cells of IPSS lower-risk MDS cases are capable of surviving under a metabolic pressure matching that of the hypoxic niche hosting normal HSC, one may conceive the use of hypoxia-activated drugs (such as the phosphate ester PR104, which is able to induce DNA cross-linking at low oxygen tension) to specifically target MDS-maintaining cells in the IPSS low/int-1 risk patients [21].

## MATERIALS AND METHODS

### Patients

BM samples were obtained from 39 patients diagnosed with different subtypes of MDS at the Haematology Unit, AOUC, Florence, Italy, after signed informed consent in accordance with the Declaration of Helsinki. MDS patient characteristics are reported in Table 1: median age, 74 years (56–89), M/F ratio, 21/18. In accordance to WHO criteria, patients were classified as: RA (6), RCMD (16), RAEB-1 (5) and RAEB-2 (8) and 5q- syndrome (4); 22 patients exhibited cytogenetic abnormalities and 16 showed a normal karyotype. According to the International Prognostic Scoring System (IPSS), 30 patients were classified as low/int-1 risk, and 9 as int-2/high risk.

### Cells and culture conditions

BMMC were separated by standard density gradient centrifugation (Lympholyte-H; Cedarlane Laboratories Ltd; Burlington, Canada). Cells were cultured in RPMI 1640 medium supplemented with 20% fetal bovine serum, 50 units/ml penicillin, 50 mg/ml streptomycin (all from EuroClone; Paington, U.K.) 50 ng/ml FLT-3, ligand, 20 ng/ml TPO, 50 ng/ml SCF and 0.5 ng/ml IL-3 (all from PeproTech; London, U.K.). Cells were incubated under standard conditions (21% O_2_) or in atmosphere at 0.1 % O_2_, in a Concept 400 anaerobic incubator (Ruskinn Technology Ltd.; Bridgend, U.K.).

### CRA assay

The CRA assay is a non-clonogenic assay developed to estimate *in vitro* the stem cell potential of cells endowed with marrow repopulation ability *in vivo* (Figure 1). This assay estimates the power of normal hematopoietic [7–9] or leukemic [13, 15–18] cells, freshly rescued from donor or subjected to an experimental treatment *in vitro*, to expand in treatment-free, growth-permissive cultures. The CRA assay represents a simple and economic method to detect *in vitro* short term-repopulating hematopoietic stem cells (STR-HSC). In the study reported here, cells incubated in hypoxia for 10 days (“selection” primary liquid culture - LC1) were transferred to fresh medium supplemented with 50 ng/ml SCF, 100 ng/ml G-CSF, 20 ng/ml IL-6 and 10 ng/ml IL-3 (“expansion” secondary liquid culture - LC2) and incubated in normoxia. To measure LC2 repopulation, cells were counted daily by trypan blue exclusion.

### CFA assay

Cells from liquid cultures incubated in hypoxia for 10 days were replated (120.000 cells/ml) in semisolid medium (MethoCult H4100; Stem Cell Technologies; Vancouver, Canada) and incubated for 14 days in the presence of the same cytokine cocktails used for the LC2 of CRA assay (see above). GEM, GM and E colonies were scored.

### Cytogenetic and FISH analysis

Cytogenetic and FISH studies were performed according to the standard methods used in our laboratory. Cultures were incubated for 48 hours at 37°C. G-banding was performed on slides kept at 60°C overnight and then stained with Wright's solution. A minimum of 20 metaphases were analyzed and described according to the International System for Human Cytogenetic Nomenclature 2013 [22]. Fluorescent *in situ* hybridization (FISH) was performed with α-satellite DNA probes for chromosomes 8 and Y (CEP8 and CEPY; Vysis; Abbott Park, IL, U.S.A.) and locus-specific probes for 7q31 and 5q31, 20q12 (LSI D7S522/CEP7, LSI EGR1 and LSI D20S108; Vysis). At least 200 nuclei were analyzed; we considered that there was a chromosomal gain when the percentage of cells with trisomy was more than 5% and a chromosomal loss when more than 10% of cells presented the anomaly.

### NGS methodology

We performed mutation analysis by NGS of 8 cases included in the study. SureDesing (Agilent Technologies; Santa Clara, CA, U.S.A.) was used to design a custom enrichment of the candidate genes: *ASXL1, EZH2, TP53, SF3B1, U2AF1, SRSF2, TET2, DNMT3A, ETV6, RUNX1, NPM1, FLT3, CBL, SETBP1, CSF3R, CEBPA, IDH1, IDH2, JAK2, MPL, CARL, NRAS.* Genes were selected after a manually curated literature screening of the most commonly mutated genes in MDS. Library preparation was performed using the HaloPlex target enrichment protocol (Agilent Technologies). The genomic DNA input for amplicon library preparation was 225 ng for each sample according to manufacturer's instructions. All sample libraries were equimolarly pooled and sequenced on the MiSeq Sequencer (Illumina; San Diego, CA, U.S.A.) with a default 150 bp paired-end reads protocol, according to the manufacturer's instructions.

### Flow cytometry

BMMC at time 0 and after 10–13 days of incubation in hypoxia or normoxia were washed and suspended in 100 ul of BD Stain Buffer (BD Biosciences; Franklin Lakes, NJ, U.S.A.), according to the manufacturer's instructions. Cells were incubated with 7-Amino-Actinomycin D (7-AAD)- Viability Dye (Beckam Coulter; Brea, CA, U.S.A.) allowing discrimination of viable from non viable cells, and then incubated with monoclonal antibodies directed to surface proteins: allophycocyanin (APC)-conjugated human CD133 (Miltenyi Biotec; Bergisch Gladbach, Germany), phycoerythrin (PE)-conjugated human CD117 (clone M-A712; BD Biosciences), PE(CY7)-conjugated human CD38 (clone HIT-2; BD Biosciences), fluorescein (FITC)-conjugated human CD34 (clone 8G12; BD Biosciences), APC(H7)-conjugated human CD45 (clone 2D1; BD Bosciences). Mouse-derived cells after xenotransplantation were incubated with peridinin-chlorophyll (PerCP)-conjugated murine CD45 (clone 30-F11; BD Biociences). Approximately 20000 events were collected for each sample using a FACSCanto analyzer (BD Biosciences) and data were processed using BD FacsDiva software.

### *In vivo* experiments

All *in vivo* experiments were performed at the Laboratory of Genetic Engineering for the Production of Animal Models (LIGeMA) at the Animal Facility of Università degli Studi di Firenze. Eight-week-old *NOD/SCID beta 2 null* mice were subjected to total body irradiation with a single 250cGy dose 24 hours before transplantation of human cells. Patient-derived BMMC rescued from hypoxia-incubated cultures were resuspended at 1 × 10^6^ cells/100 ul final concentration and then injected via the tail vein. Every two weeks from transplant, blood was drawn from the retro-orbital plexus and collected into EDTA-containing tubes, red blood cells were haemolysed, and mononuclear cells washed with PBS. At the scheduled end of experiment or when the mice were moribund, femora, tibiae and spleens were collected. Cells were flushed from femora and tibiae using 23G needles. Spleen was minced and passed through 70 μm filters. Marrow and spleen cells were washed in haemolytic buffer and resuspended in PBS. The presence of CD45+ human cells in PB, BM and spleen of transplanted mice was evaluated by flow cytometry. Survival of mice for at least 28 days after transplantation was considered and indicator of successful engraftment, together with the presence of ≥0.2% of human CD45+ cells in marrow or spleen [23].

## SUPPLEMENTARY MATERIALS FIGURE



## Abbreviations

AML: Acute Myeloid Leukemia
BM: Bone marrow
BMMC: Bone marrow mononuclear cells
CFA: Colony formation ability
CRA: Culture repopulating ability
FISH: Fluorescent *in situ* hybridization
HSC: Hematopoietic stem cells
MDS: Myelodysplastic syndromes
LC1: Primary liquid cultures
RA: Refractory anemia
RAEB-1: Refractory anemia with excess of blasts 1
RAEB-2: Refractory anemia with excess of blasts 2
RCMD: Refractory cytopenia with multilineage dysplasia
LC2: Secondary cultures
STR-HSC: Short term-repopulating hematopoietic stem cells
WHO: World health organization
