# Supplementary material for: Severe hypoxia selects hematopoietic progenitors with stem cell potential from primary Myelodysplastic syndrome bone marrow cell cultures
Source: Oncotarget. 2018 Jan 24;9(12):10561–71. doi: 10.18632/oncotarget.24302 (PMC5828219; doi:10.18632/oncotarget.24302)
Supplement: Supplementary file 1 [file oncotarget-09-10561-s001.pdf]

Severe hypoxia selects hematopoietic progenitors with stem cell potential from primary Myelodysplastic syndrome bone marrow cell cultures

SUPPLEMENTARY MATERIALS

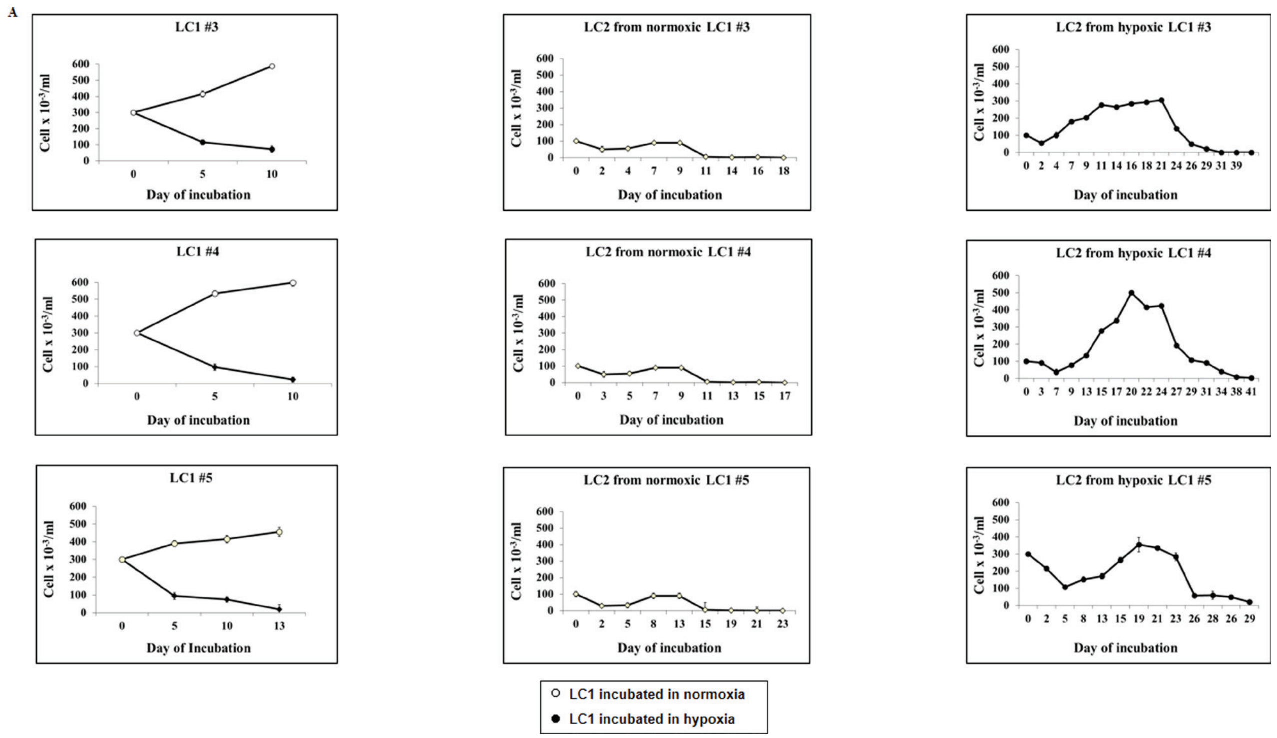

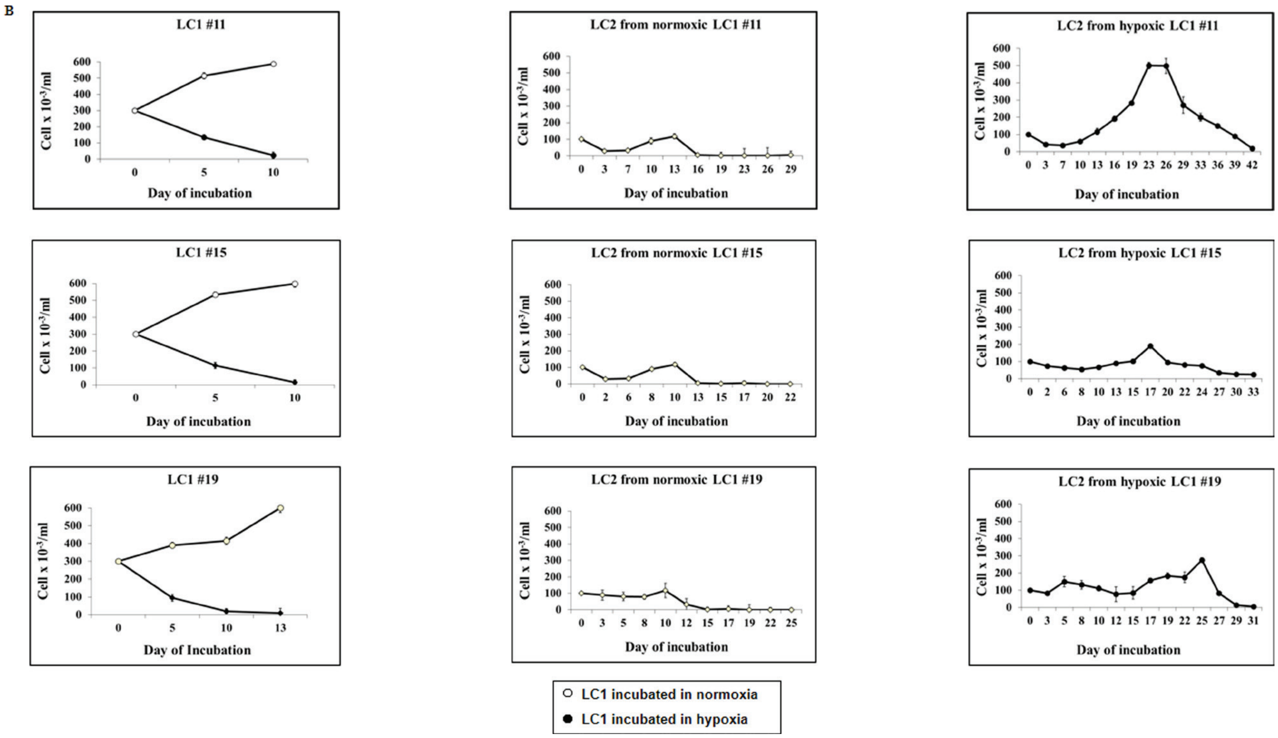

C

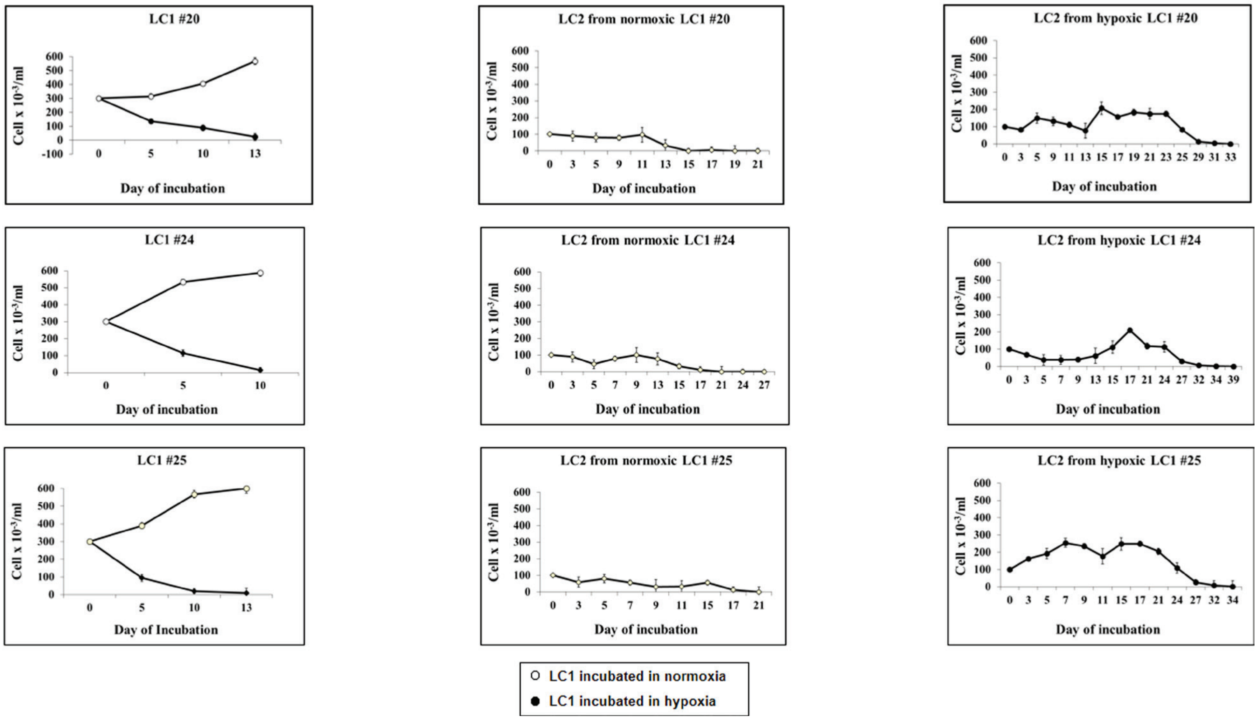

LC1 #20
LC2 from normoxic LC1 #20
LC2 from hypoxic LC1 #20
LC1 #24
LC2 from normoxic LC1 #24
LC2 from hypoxic LC1 #24
LC1 #25
LC2 from normoxic LC1 #25
LC2 from hypoxic LC1 #25
Cell x 10-3/ml
Day of incubation
Day of Incubation
LC1 incubated in normoxia
LC1 incubated in hypoxia

D

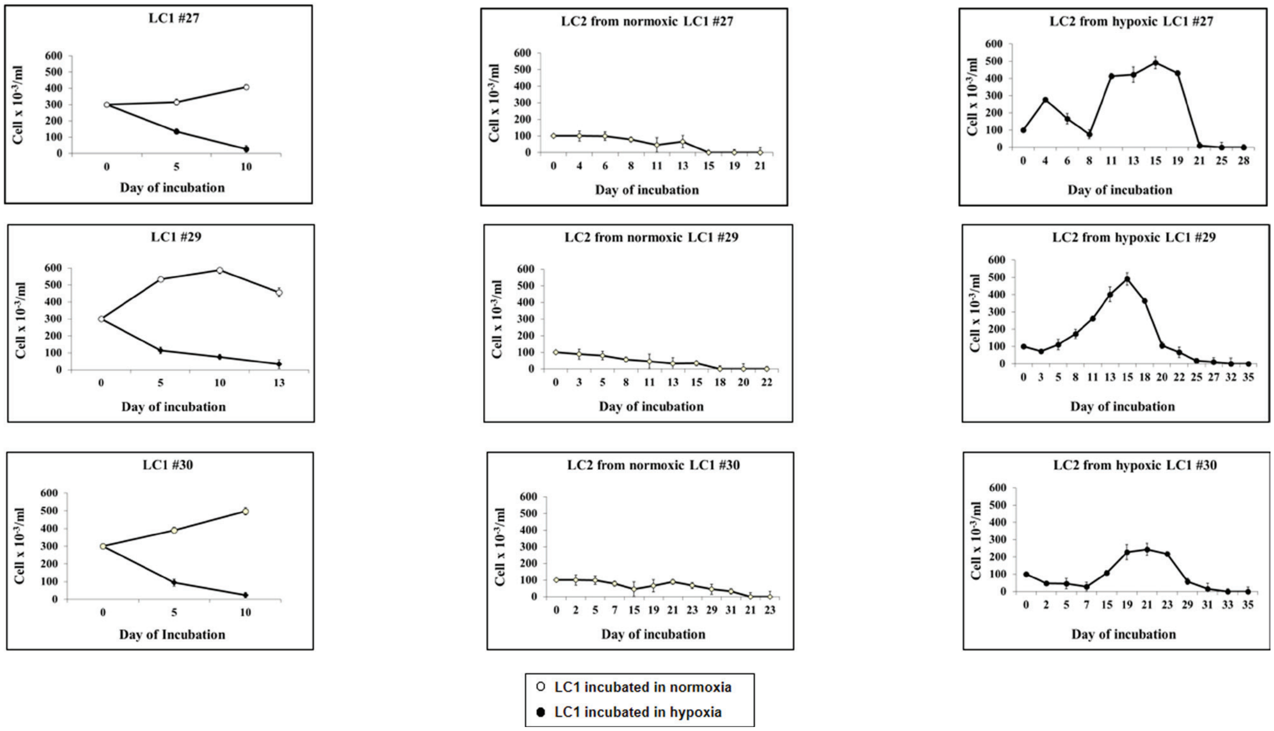

LC1 #27
LC2 from normoxic LC1 #27
LC2 from hypoxic LC1 #27
LC1 #29
LC2 from normoxic LC1 #29
LC2 from hypoxic LC1 #29
LC1 #30
LC2 from normoxic LC1 #30
LC2 from hypoxic LC1 #30
Cell x 10-3/ml
Day of incubation
Day of Incubation
LC1 incubated in normoxia
LC1 incubated in hypoxia

E

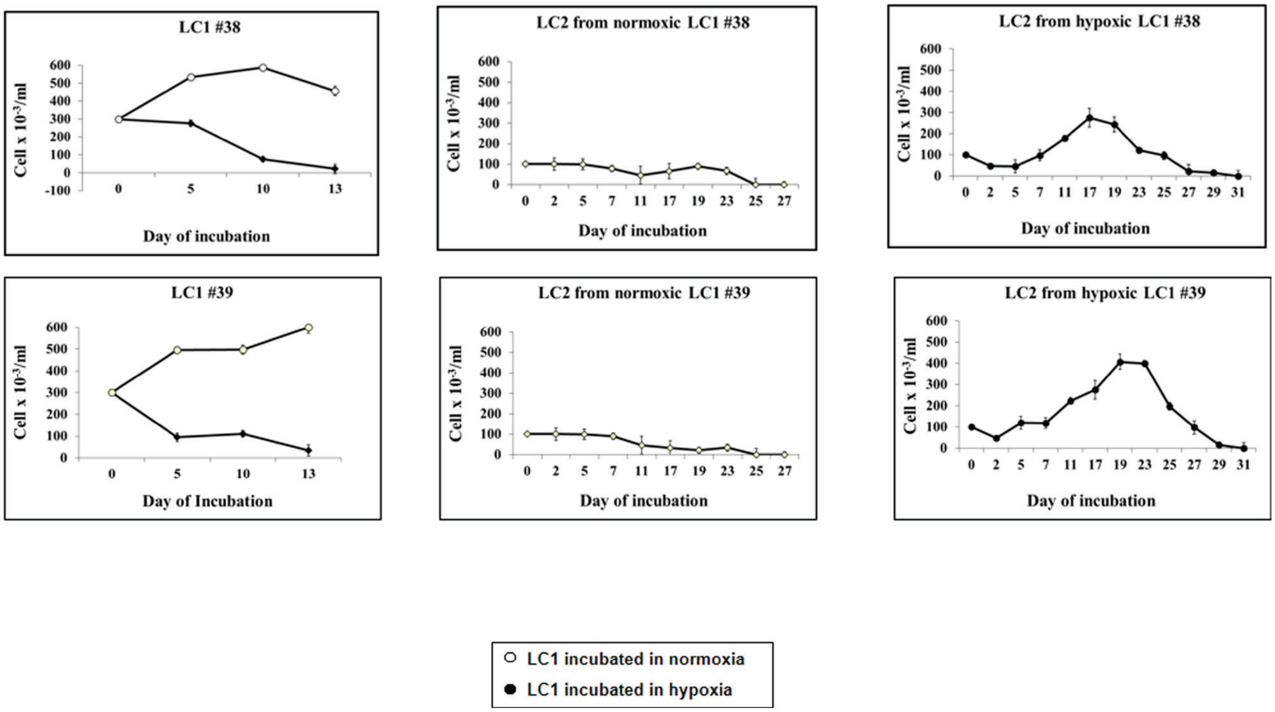

Supplementary Figure 1A–1E: Effects of incubation in hypoxia or normoxia on total cell number and stem cell potential of MDS cases positive for CRA. BMMC were explanted from 14 MDS patients (see Table 1 and Table 2). Cells were incubated in low oxygen or in air (LC1) for 10–13 days (left panels) and then transferred to secondary cultures incubated in air (LC2) to measure stem cell potential of LC1 cells by CRA assay (cells from normoxic LC1, middle panels; cells from hypoxic LC1, right panels).
